# Active-Site Oxygen Accessibility and Catalytic Loop Dynamics of Plant Aromatic Amino Acid Decarboxylases from Molecular Simulations

**DOI:** 10.1021/acs.biochem.4c00144

**Published:** 2024-07-15

**Authors:** Yitao Gou, Tianjie Li, Yi Wang

**Affiliations:** Department of Physics, The Chinese University of Hong Kong, Shatin, New Territories, Hong Kong, China

## Abstract

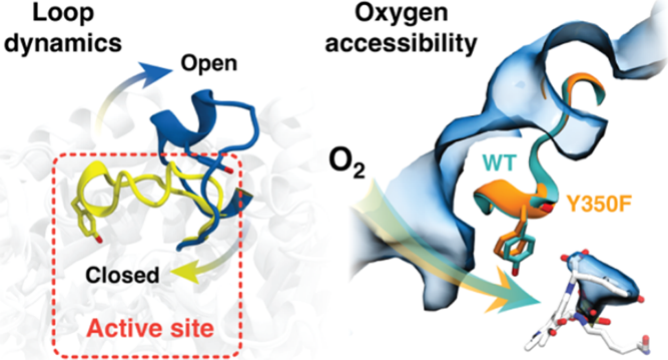

Aromatic amino acid decarboxylases (AAADs) are pyridoxal-5′-phosphate (PLP)-dependent enzymes that catalyze the decarboxylation of aromatic amino acid l-amino acids. In plants, apart from canonical AAADs that catalyze the straightforward decarboxylation reaction, other members of the AAAD family function as aromatic acetaldehyde synthases (AASs) and catalyze more complex decarboxylation-dependent oxidative deamination. The interconversion between a canonical AAAD and an AAS can be achieved by a single tyrosine-phenylalanine mutation in the large catalytic loop of the enzymes. In this work, we report implicit ligand sampling (ILS) calculations of the canonical l-tyrosine decarboxylase from *Papaver somniferum* (*Ps*TyDC) that catalyzes l-tyrosine decarboxylation and its Y350F mutant that instead catalyzes the decarboxylation-dependent oxidative deamination of the same substrate. Through comparative analysis of the resulting three-dimensional (3D) O_2_ free energy profiles, we evaluate the impact of the key tyrosine/phenylalanine mutation on oxygen accessibility to both the wild type and Y350F mutant of *Ps*TyDC. Additionally, using molecular dynamics (MD) simulations of the l-tryptophan decarboxylase from *Catharanthus roseus* (*Cr*TDC), we further investigate the dynamics of a large catalytic loop known to be indispensable to all AAADs. Results of our ILS and MD calculations shed new light on how key structural elements and loop conformational dynamics underlie the enzymatic functions of different members of the plant AAAD family.

## Introduction

Aromatic amino acid decarboxylases (AAADs) are pyridoxal-5′-phosphate (PLP)-dependent enzymes of ancient evolutionary origin that catalyze the decarboxylation of aromatic l-amino acids.^1−3^ In plants, these enzymes are involved in the biosynthesis of secondary metabolites,^4^ i.e., compounds that are not essential for growth or development but render plants competitive in their own environment. While mammals have only a single AAAD that catalyzes the decarboxylation of aromatic amino acids, the plant AAAD family has undergone extensive evolutionary diversification, giving rise to a series of paralogous enzymes with different substrate preferences and catalytic mechanisms.^3^ For instance, tryptophan decarboxylases (TDCs) and tyrosine decarboxylases (TyDCs), known as canonical AAADs, catalyze the straightforward decarboxylation of tryptophan (Trp) and tyrosine (Tyr), respectively. Two other members of the plant AAAD family, phenylacetaldehyde synthases (PAASs) and 4-hydroxyphenylacetaldehyde synthases (4HPAASs), known as aromatic aldehyde synthases (AASs), catalyze the more complex decarboxylation-dependent oxidative deamination^5^ of phenylalanine (Phe) and Tyr, respectively.

**Figure 1:**
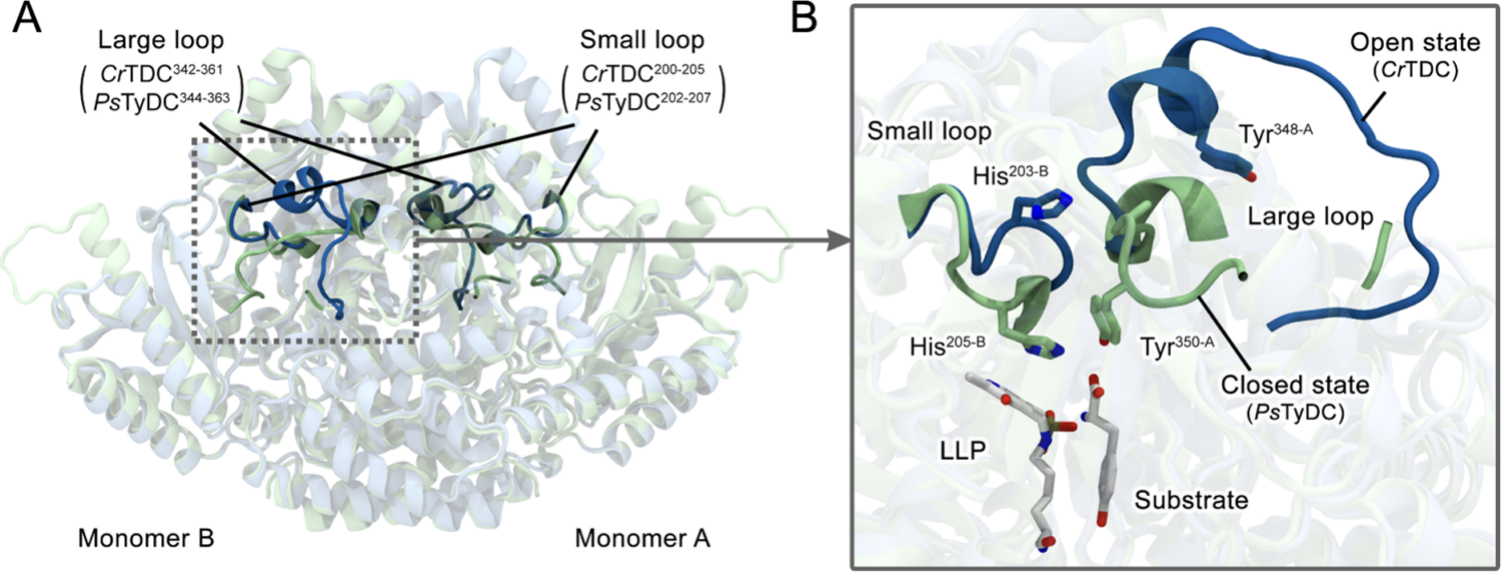
Crystal structures of *Cr*TDC and *Ps*TyDC.^14^ (A) Superimposed crystal structures of *Cr*TDC (blue) and *Ps*TyDC (lime). (B) Magnified snapshot showing different conformations of the large loop and the small loop in the crystal structures of *Cr*TDC (blue) and *Ps*TyDC (lime). For clarity, only the substrate tyrosine of *Ps*TyDC is shown, while the substrate tryptophan of *Cr*TDC was omitted.

PLP-dependent enzymes primarily participate in the biosynthesis of amino acids and amino acid-derived metabolites.^6^ Unrivaled in the versatility of reactions they catalyze, these enzymes are estimated to collectively account for ∼4% of all enzyme activities.^7^ Despite their diverse reaction types, PLP-dependent enzymes employ a unified mechanism where the cofactor acts to stabilize the negative charge on the substrate C_α_ atom developed during the reaction transition state.^6^ Specifically, the PLP cofactor is initially linked to the ε-amino group of a conserved active-site lysine, forming an internal aldimine (LLP, see Figure 1); upon binding of an amine-containing substrate, LLP undergoes a Schiff base exchange reaction (transimination), during which a substrate-PLP linkage replaces the enzyme-PLP one, and the resulting external aldimine serves as a common intermediate for all PLP-dependent reactions. At this point, divergence in reaction types begins.^1,8^ For decarboxylases, the bond between the C_α_ atom and the carboxylate group is cleaved, producing CO_2_ and a carbanion, with the negative charge of the latter stabilized by PLP in the form of a quinonoid intermediate. In canonical AAADs, this quinonoid intermediate is then protonated to yield the monoamine product and a regenerated LLP; in AASs; however, an oxygen molecule attacks the quinonoid intermediate and eventually produces the aromatic acetaldehyde product, along with ammonia and hydrogen peroxide.^5,9−11^

Mutagenesis and structural studies have established the critical role of a conserved tyrosine in AAADs.^11,12^ Upon mutation of this tyrosine to a phenylalanine, canonical plant AAADs are converted into AASs. Conversely, a Phe-to-Tyr mutation converts AASs to canonical AAADs. Notably, the decarboxylase activity of the resulting mutant is even faster than the aldehyde synthase activity of the wild-type (WT) AAS.^11^ A single Tyr-to-Phe mutation has also been reported to turn the mammalian AAAD l-Dopa decarboxylase (DDC) and the human histidine decarboxylase (HDC) into acetaldehyde synthases.^12,13^ Such a dramatic impact of a single amino acid has been attributed to the role of tyrosine as a proton donor to the reaction intermediate: this key tyrosine in decarboxylases acts to protonate the quinonoid intermediate; when it is replaced by a phenylalanine that cannot serve as a proton donor, molecular oxygen steps in, forcing the quinonoid intermediate to undergo oxidative deamination.

The importance of the key tyrosine and the additional role played by a conserved histidine is further illustrated in a recent work that combines structural and functional studies with molecular dynamics (MD) simulations of plant AAADs.^14^ In this work, X-ray crystal structures of four plant AAADs were resolved, including *Catharanthus roseus* TDC (*Cr*TDC), *Papaver somniferum* TyDC (*Ps*TyDC), *Arabidopsis thaliana* PAAS (*At*PAAS), and *Rhodiola rosea* 4HPAAS (*Rr*4HPAAS). All four enzymes were found to be homodimers with two symmetric active sites located at the dimer interface (Figure 1). The key Tyr residue (Y348 in *Cr*TDC or Y350 in *Ps*TyDC) was located on a large loop that had been missing in X-ray crystal structures of mammalian DDCs with the exception of the recently resolved human aromatic amino acid decarboxylase.^15^ In *Cr*TDC, this loop adopted an open conformation, exposing the active site. In *Ps*TyDC, this loop adopted a closed conformation that sealed the active site, although a small portion (residues 354–359) of the loop was unresolved. Another important structural difference between the two canonical AAADs involved a small loop harboring a conserved histidine (H203 in *Cr*TDC or H205 in *Ps*TyDC, see Figure 1). In *Cr*TDC, this small loop was rotated outward, while in *Ps*TyDC, it adopted a conformation that enabled the aforementioned histidine to form a π–stacking interaction with LLP’s pyridine ring. Our previous MD simulations spanning hundreds of nanoseconds^14^ showed that initiated from the *Cr*TDC-crystal structure in the open state, the large loop could undergo a swing motion that brought the key tyrosine within hydrogen bonding distance of the small-loop histidine. Based on the above structural and dynamic data as well as enzyme assays and transgenic yeast expressing WT, Y350F, or H205N mutant of *Ps*TyDC, the small-loop histidine was identified as an important partner of the large-loop tyrosine, whereby the former amino acid facilitated the latter’s proton transfer to the quinonoid intermediate.^14^

While the above study has shed new light on the mechanistic basis of the functional divergence within the AAAD family, much remains to be learned about these enzymes. For instance, the Tyr-to-Phe mutation has been suggested to produce a larger cavity due to the absence of the hydroxyl group in phenylalanine, which may in turn facilitate the entrance of molecular oxygen into the active site.^14^ Given that the presence of O_2_ at the active site is a prerequisite of AASs’ oxidative deamination activity, it is conceivable that AAADs and AASs may have utilized the different sizes and hydrophobicities of the two amino acids to achieve differential oxygen accessibility within their active sites. Whether this is indeed the case, however, cannot be solely determined from static structural information. Another feature of the plant AAAD family that remains to be further investigated is the dynamics of their large loops that harbor the conserved tyrosine. Our previous work on *Cr*TDC demonstrated considerable flexibility of this large loop, revealing its transition from an open to a semiclosed state in ∼500 ns MD simulations. However, the limited time span of these calculations and the lack of counterpart simulations initiated from the closed state hindered sampling of the loop conformational space and estimation of the characteristic correlation time of loop movement. Here, we report implicit ligand sampling (ILS) calculations on the closed-state *Ps*TyDC in both the wild-type enzyme and its Y350F mutant. Contrary to the aforementioned hypothesis, the three-dimensional (3D) free energy profiles from ILS performed on both apo- and holo-forms of *Ps*TyDC reveal similar pathways and comparable energy barriers of molecular oxygen entering the active sites of the WT, canonical AAAD, and its Y350F mutant that has been converted into an AAS. We further report microsecond MD simulations of *Cr*TDC initiated from both the open and closed states of the large loop, demonstrating a considerably broader sampling of loop conformations than those recovered from our previous, hundreds of nanoseconds simulations. Based on these results, we discuss the impact of the key tyrosine/phenylalanine on differentiating the reaction specificities of canonical AAADs and AASs, the correlation between loop secondary structure and dynamics, and sampling consideration for future modeling studies of the plant AAAD family.

## Methods

### Protein Structure Preparation

**Figure 2:**
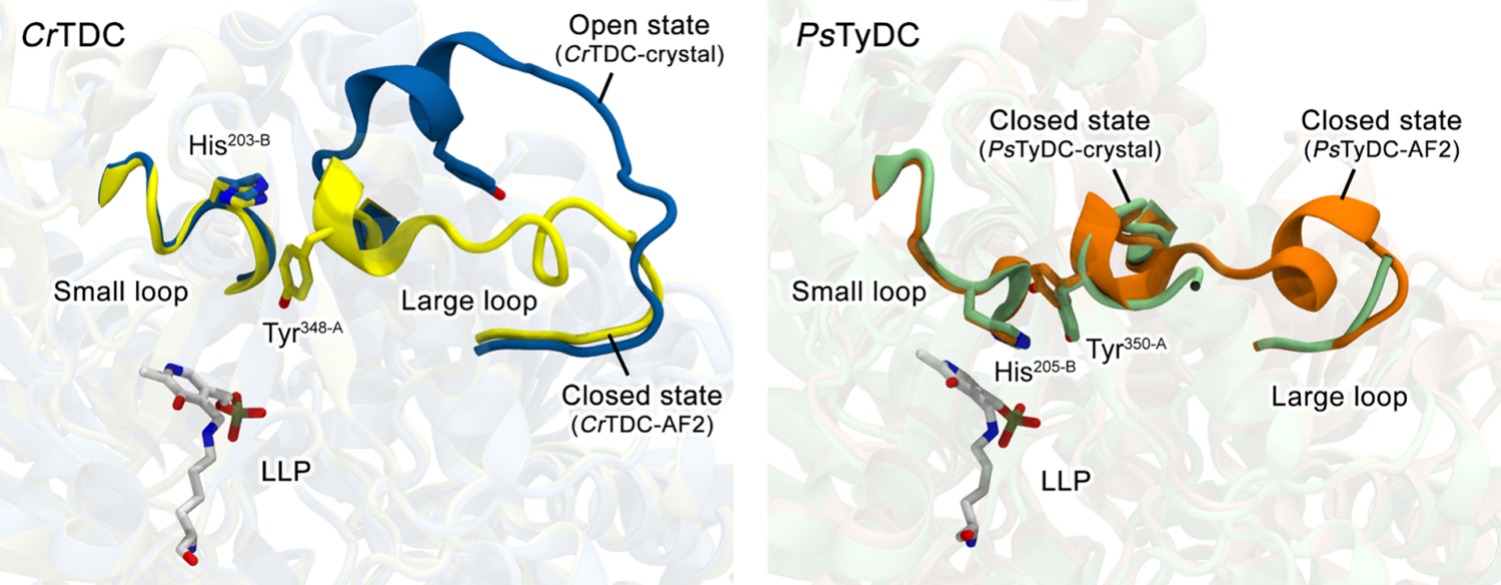
AlphaFold2-predicted structures of *Cr*TDC (*Cr*TDC-AF2, yellow) and *Ps*TyDC (*Ps*TyDC-AF2, orange) reveal similar closed conformations of their large loops. Crystal structures of the two enzymes with LLP are shown for reference (*Cr*TDC-crystal, blue; *Ps*TyDC crystal, lime).

The crystal structures of *Cr*TDC and *Ps*TyDC were obtained from the Protein Data Bank (PDB codes: 6eew, 6eem(14)). The large catalytic loop, in which the key tyrosine is located, adopts an open conformation in *Cr*TDC (residues 342–361) and a closed conformation in *Ps*TyDC (residues 344–363), where a small segment of the *Ps*TyDC loop (residues 354–359) is missing. To construct the closed-state *Cr*TDC, we turned to AlphaFold2 (AF2),^16^ which was also used to model the missing segment in the *Ps*TyDC loop. As shown in Figure 2, apart from the region around the missing loop segment, the AF2 prediction of *Ps*TyDC is nearly indistinguishable from its crystal structure. The predicted missing segment, which forms a short α-helix, resembles the corresponding segment in the crystal structure of human HDC (data not shown). For *Cr*TDC, the AF2-predicted closed conformation is highly similar to that of the *Ps*TyDC crystal structure (Figure 2). To construct MD simulation systems, monomeric structures of *Cr*TDC and *Ps*TyDC obtained from AlphaFold Protein Structure Database^17^ were aligned to their crystal structures in order to produce the corresponding homodimers. A *Cr*TDC residue distant from the active site, Gly401, is replaced by an alanine in the crystal structure (PDB code: 6eew). As the crystal and AF2 structures were nearly indistinguishable except for the large loop, the *Cr*TDC-crystal model was built with its large loop from the crystal structure and the rest of the protein from the AF2 structure so that Gly401 was consistently adopted in both of our *Cr*TDC models. While most residues were found in their default protonation states, several were modeled in their neutral forms based on PROPKA-3.1^18,19^ results. These residues are Asp268-A/B, Asp397-A/B, Lys208-A/B, and Glu169-A for *Cr*TDC, and Asp270-A/B and Lys379-A/B for *Ps*TyDC. The single amino acid mutants (*Ps*TyDC-Y350F) were constructed using PyMOL-Mutagenesis Wizard.^20^ Unless otherwise stated, all simulation systems were constructed with the proteins in their apo-states, i.e., without the substrate or PLP cofactor.

### MD Simulations and Analysis

To prepare for O_2_ accessibility calculation via ILS, five replicas of 100 ns plain MD simulations of apo-*Ps*TyDC were first carried out for the WT protein or the Y350F mutant using NAMD2.14^21^ and the CHARMM36 force field.^22^ Apart from apo-*Ps*TyDC, five replicas of 100 ns plain MD simulations were also performed for a holo-*Ps*TyDC in complex with both the internal aldimine LLP and the substrate Tyr. The protonation states of LLP and Tyr follow that of system 1 in our previous work:^14^ briefly, the PLP aldimine is known to undergo an enolimine-ketoenamine tautomerism, with a proton transferred between its phenolic oxygen (enolimine) and aldimine nitrogen (ketoenamine).^23−25^ In enzymes with a strategically placed acidic residue (Asp289 in *Ps*TyDC), a strong hydrogen bond involving the pyridine nitrogen favors the ketoenamine form of LLP by shifting the proton in its intramolecular hydrogen bond from the phenolic oxygen to the aldimine nitrogen.^23^ However, unlike the enolimine form that has a good analogy with existing compounds in the latest CHARMM General Force Field (CGenFF, version 4.6) as reflected in its low penalty values of predicted parameters, the ketoenamine form lacks such a good analogy (data not shown) and the protein environment that stabilizes this tautomer cannot be readily included in its parametrization protocols. For this reason, we adopted the enolimine form of LLP in the ILS calculations, along with a neutral pyridine nitrogen and a neutral Asp289 (Figure S1A).^23^ The substrate Tyr is modeled in its zwitterionic form, mimicking a state prior to the formation of the Michaelis complex.^26^ The enolimine form of LLP was parametrized using CGenFF^27−29^ with the conjugated lysine 321 atom slightly adjusted in its atomic partial charge to render the overall charge of the molecule an integer. Finally, five replicas of 100 ns plain MD simulations were also performed for a second holo-*Ps*TyDC in complex with the external aldimine (Figure S1B) that has been crystallized in one of its monomers.^14^ Parameters for this external aldimine were again generated by CGenFF and its high-penalty charges and dihedrals were optimized using the Force Field Toolkit plugin of VMD.^30^ Final parameters of LLP and external aldimine used in our simulations along with their corresponding structures are available at GitHub repository: https://github.com/TianjieLi-Jason/PsTyDC.git. The apo- and both holo-*Ps*TyDC systems were solvated in a water box with 0.15 M NaCl to neutralize the systems, resulting in a total of ∼74,000 atoms. A 12 Å cutoff was applied for nonbonded interactions, while long-range electrostatics were computed using the particle mesh Ewald (PME) method^31,32^ with a grid spacing of 1 Å. The temperature was maintained at 300 K by Langevin dynamics and the pressure was kept at 1 atm by Nosé–Hoover Langevin piston.^33,34^ To maintain the closed-state structure of *Ps*TyDC, positional restraints with a force constant of 5 kcal/(mol·Å^2^) were applied to the C_α_ atoms of its large catalytic loop as well as the α-helices and β-sheets of the rest of the protein. In holo-*Ps*TyDC, the positional restraints were also applied to the C_α_ atoms of LLP and Tyr as well as the external aldimine. All simulation trajectories were written every 10 ps, which were then utilized in subsequent ILS calculations.

All *Cr*TDC simulations were performed using GROMACS 2022.5^35^ with the CHARMM36 force field.^22^ To distinguish simulations initiated from the loop-open conformation of the *Cr*TDC-crystal structure and the loop-closed conformation of its AF2 prediction, hereon we refer to these two sets of simulations by their initial structures (*Cr*TDC-crystal and *Cr*TDC-AF2, respectively). Each initial dimer structure was solvated in a dodecahedron box with TIP3P water, and 0.05 M NaCl was added to neutralize the system (Figure S2), resulting in a total of ∼120,000 atoms. After a 2000-step energy minimization, the systems underwent a 1 ns NVT and a 1 ns NPT equilibration successively. Subsequent 8 μs production simulations were carried out in the NPT ensemble in two replicas for *Cr*TDC-crystal and *Cr*TDC-AF2, respectively, resulting in a total of 32 μs simulations for these two systems. The temperature was coupled to 300 K using velocity rescaling,^36^ while the pressure was coupled to 1 bar using the c-rescaling method.^37^ Bonds involving hydrogen atoms were constrained using the LINCS algorithm.^38,39^ Nonbonded interactions were cut off at 12 Å, and the PME method^31,32^ was applied for electrostatic interactions. To analyze loop conformations from MD trajectories, the longest principal axis of the *Cr*TDC dimer was aligned with the *z* axis using VMD.^40^ The minimum distance *d* between atoms of Tyr348 and His203 in the same active site was computed by using GROMACS (*gmx mindist*). The principal component analysis (PCA) was performed on the C_α_ atoms of the large loop by using *gmx covar* and *gmx anaeig*. Clustering analysis was conducted on combined trajectories of aligned *Cr*TDC monomers using the root-mean-square deviation (RMSD)-based GROMOS method with a cutoff of 6.2 Å. In all of the analysis regarding loop dynamics, the two monomers in a *Cr*TDC dimer were treated equivalently. Secondary structural contents of the large catalytic loop were calculated using the DSSP program (version 4).^41,42^ The total percentage of residues classified as α-helix or 3-helix was taken as the loop’s α-helical content.

### Implicit Ligand Sampling (ILS)

Instead of simulating actual oxygen migration events, the ILS approach makes use of the small size of O_2_ to conduct a single-step free energy perturbation (FEP) in a postprocessing manner, yielding a complete 3D free energy map of the gas molecule within a given system.^43,44^ Specifically, the 3D potential of mean force (PMF) of a gas molecule is estimated as
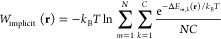
1where Δ*E*_*m*,*k*_ is the interaction energy of adding a gas molecule at position **r** with a given orientation (*k*) in simulation frame *m* (the explicit dependence of Δ*E*_*m*,*k*_ on protein and solvent coordinates as well as ligand orientation is omitted for clarity), *N* stands for the total number of simulation frames, and *C* represents the number of ligand orientations explored at each frame.^44^ The resulting 3D PMF depends on the gas molecule’s position **r** only, while its orientational degrees of freedom have been averaged out. With Δ*E*_*m*,*k*_ = 0 in a vacuum, the thus obtained free energy value represents the cost of moving the gas molecule from a vacuum to the given location. For instance, the free energy value obtained for the bulk water region corresponds to the solvation free energy of the gas molecule. The difference between the free energy value obtained within a protein cavity and this solvation free energy can then be utilized to analyze the energetic cost, if any, of transferring the gas molecule from bulk water to the given position within the protein. Further details of the ILS method can be found in the work of Cohen et al.^43,44^

The VolMap command in VMD was used to conduct the ILS calculation, where a total of *N* = 10000 snapshots were extracted from each 100 ns MD simulation of the WT or Y350F mutant of *Ps*TyDC. The ILS calculation was performed at 300 K with a grid spacing of 1 Å. The Lennard-Jones parameters for molecular oxygen were σ = 1.7 Å and ε = −0.12 kcal/mol.^43^ At each grid point, the free energy of placing an O_2_ molecule was computed by setting the parameter *orient* to 7, which generated 21 probe orientations after exploiting the C2 rotary symmetry of an oxygen molecule. Further increasing the values of parameter *orient* to 8 or 9 did not significantly improve the ILS result (data not shown). For each of the five replicas of 100 ns simulations used in ILS calculations, analysis of the two *Ps*TyDC monomers was performed separately, yielding *n* = 10 samples of O_2_ free energy barriers in either apo-, holo-(LLP + substrate), or holo-(external aldimine) *Ps*TyDC. Additionally, in each simulation replica, a region far from the protein was selected, yielding altogether *n* = 10 (five from WT and five from Y350F mutant) estimation of the free energy cost of placing O_2_ in the bulk water. Statistical significance of the difference between two systems, e.g., apo-WT and apo-Y350F mutants, was assessed from all samples of each system using a two-sample *t*-test. All reported errors represent standard deviation. The propagation of error was achieved by taking the square root of the sum of the individual errors. In addition to ILS calculation, 3D water occupancy maps were computed using the same MD simulation trajectories at a resolution of 1 Å by the VolMap plugin of VMD.

## Results

### Active-Site O_2_ Accessibility in WT and Y350F Mutant of *Ps*TyDC

Mutagenesis experiments have shown that substituting a tyrosine on its large catalytic loop by a phenylalanine is sufficient to transform a canonical TyDC into an AAS, reflecting the key role of the conserved Tyr residue.^11^ The removal of a hydroxyl group upon the Tyr-to-Phe mutation enlarges the active-site cavity, which has been hypothesized to facilitate molecular oxygen to occupy the position originally taken by this proton-donating group.^14^ Here, in order to quantify O_2_ accessibility to the active site, we determined its 3D free energy profiles in both the WT enzyme and the Y350F mutant of *Ps*TyDC via implicit ligand sampling (ILS). To comprehensively explore potential pathways utilized by O_2_ to enter the active site, ILS calculations were performed on five replicas of 100 ns simulations for each of the apo- and two holo-forms of *Ps*TyDC, with the latter in complex with either the internal aldimine LLP and substrate, or the external aldimine (Figures S1 and S3). As detailed in the Methods Section, ILS takes advantage of the relatively weak interaction between a gas molecule and its environment to conduct a single-step FEP calculation.^43^ A plain MD simulation of the solvated protein, without any O_2_, is conducted and ILS is performed in a postprocessing manner to extract the free energy cost of virtually placing a molecular oxygen at a given position of a 3D grid spanning the simulation system, i.e., treating O_2_ as an implicit ligand. The computed free energy value at each point of the 3D grid reflects the probability of finding an oxygen at that position relative to the vacuum (set to have a free energy value of 0 kcal/mol). Isosurfaces connecting points with the same free energy can be used to highlight migration pathways that the gas molecule may take to enter the protein active site from the bulk solution.

**Figure 3:**
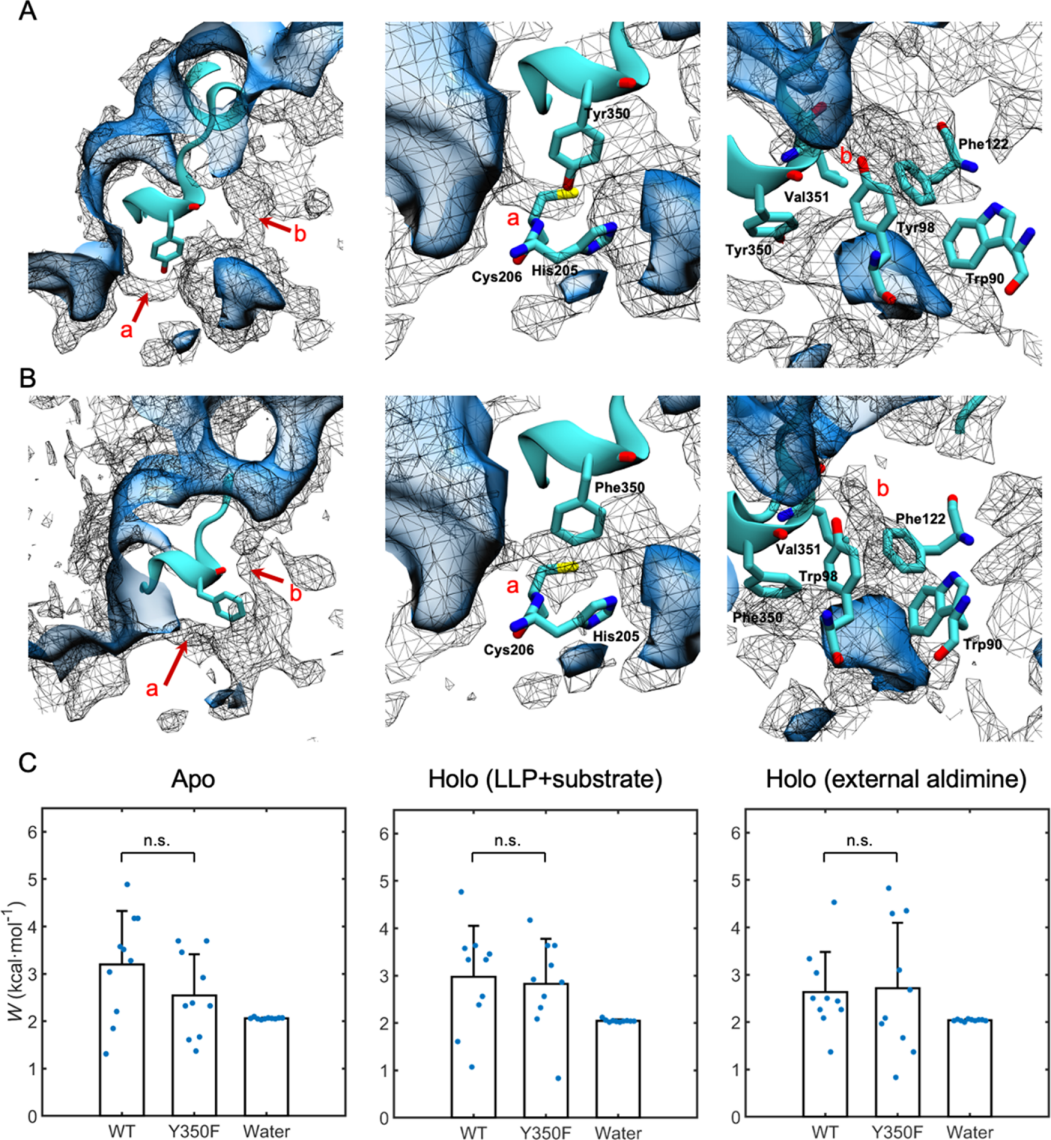
3D PMF of O_2_ calculated by ILS in the WT protein (A) and the Y350F mutant (B) of *Ps*TyDC. The PMF profiles are represented by black wireframes with energy isosurfaces at 3.9 kcal/mol (WT) and 2.6 kcal/mol (Y350F), respectively. The two pathways (**a**, **b**) leading to the protein active site from bulk water are indicated by red arrows, with residues lining each pathway being highlighted. Common to all images are the protein surface (blue cavity), the catalytic large loop (cyan), and the key Tyr350/Phe350 residue (licorice). (C) Free energy values of O_2_ in water and at barrier locations along their migration pathways (see (A, B)) from the exterior of the protein to the active site in apo-*Ps*TyDC, holo-*Ps*TyDC with LLP and substrate, and holo-*Ps*TyDC with external aldimine, respectively. The difference between WT and Y350F lacks statistical significance at the 0.05 level (denoted by ns) in all three simulation systems.

Our ILS calculations revealed two major entrance pathways of oxygen into the active sites of both the WT protein and the Y350F mutant of *Ps*TyDC, the representative snapshots of which are shown in Figure 3A,B. One of the two pathways (labeled **a**) is near the side chain of the key Tyr350 or its Phe350 substituent in the mutant protein, while the other (labeled **b**) is on the “back” of the first pathway and exits the protein surface near the loop residue Val351. By comparing the maximum free energy value along each pathway, we obtained the minimum free energy barrier that an oxygen molecule must overcome in order to take the “easier” path of the two and enter the active site. From five replicas of 100 ns plain MD simulations of each apo- or holo-*Ps*TyDC dimers, we obtained 10 ILS maps that yielded the energy barriers as scattered dots in Figure 3C. Their mean and standard deviation are listed in Table S1, along with the O_2_ solvation free energy computed for bulk water from the corresponding simulations. Based on the two-sample *t*-test (see the Methods Section), no statistically significant difference (at the 0.05 level) is found in any of the WT vs Y350F mutant comparison, i.e., they present similar barriers to O_2_ permeation in apo- as well as both holo-forms investigated here (Figure 3C). Our computed O_2_ solvation free energy (2.05 ± 0.02 kcal/mol) is in good agreement with previous ILS calculations (1.97 ± 0.02 kcal/mol)^43^ performed at the same temperature (300 K) and comparable with the experimental value measured at 20 °C (1.78 kcal/mol).^45^ Subtracting this computed solvation free energy from the ILS results of a given *Ps*TyDC dimer yields the energy barrier that an O_2_ molecule must overcome in its migration from bulk solution into the protein’s active site. The thus obtained barriers of the WT *Ps*TyDC and Y350F mutant are 0.89 ± 1.02 and 0.65 ± 1.06 kcal/mol, respectively, the difference between which is again insignificant according to the two-sample *t*-test. Furthermore, although these barrier heights have statistically significant (*P* < 0.05) differences from zero, their values are comparable to the thermal energy *k*_B_*T* (∼0.60 kcal/mol at 300 K), suggesting that they can be readily overcome by a diffusing oxygen molecule. The above results indicate that molecular oxygen encounters similarly small barriers entering the active sites of the WT and Y350F *Ps*TyDC. In other words, to this gas molecule, both enzyme active sites are nearly as readily accessible as the bulk solution.

In both the WT protein and Y350F mutant, neither pathway **a** nor **b** consistently dominates over the other to always represent the “easier” path for O_2_ migration. This result and the aforementioned small energy barrier values suggest that the “breathing motion” of *Ps*TyDC readily opens an entrance path, one way or the other, for the small gas molecule. Further analysis of the 3D O_2_ free energy profiles identifies residues lining each of the two major pathways: pathway **a** is guarded by the key tyrosine (or its phenylalanine substitute), as well as His205 and Cys206. The narrowest segment of pathway **b** is lined by a number of aromatic residues, including Trp90, Phe122, and Tyr98 as well as the hydrophobic Val351 that neighbors Tyr350. Overall, the absence of a large energy barrier against the entrance of oxygen to the *Ps*TyDC active site may be attributed to the relatively short length of pathway **a** and the largely hydrophobic nature of pathway **b**. Comparison with the 3D water occupancy maps obtained from the same simulations suggests that these two pathways are at least partially solvated, albeit to different extent (Figure S3).

### Dynamics of the Large Catalytic Loop from *Cr*TDC

In order to serve as the proton donor to the quinonoid intermediate, the key tyrosine in canonical AAADs must be positioned near the substrate for which the large catalytic loop needs to adopt its closed conformation. In the crystal structure of *Cr*TDC, the large loops in both monomers are in an open state. Previously, we conducted 36 sets of 100 ns MD simulations (with one of them extended to ∼500 ns) on this structure to explore the initial transition of the loop from an open to a semiclosed conformation, which was found to be independent of the protonation states of the substrate and LLP.^14^ However, the relatively short simulation time and the sole initial structure from which the simulations were launched limited the sampling of loop dynamics. In this work, we performed multimicrosecond MD simulations of apo-*Cr*TDC initiated either from its open state revealed by the crystal structure (*Cr*TDC-crystal) or from a closed state predicted by AlphaFold2 (*Cr*TDC-AF2) that resembles the crystal structure of the closed-state *Ps*TyDC (Figure 2). As our goal is to extract characteristic structural and dynamic features in the large loop’s exploration of its conformational space (as opposed to being locked in a given state), the apo-form of *Cr*TDC in the absence of substrate and LLP was employed, since the loop was found to be highly mobile in the apo-form from our previous study.^14^ Specifically, two replicas of 8 μs MD simulations were initiated with either the open (*Cr*TDC-crystal) or closed (*Cr*TDC-AF2) state of the loop as its initial structure. With both monomers in the homodimeric enzyme treated equivalently, these simulations collectively provided 64 μs trajectories for the large catalytic loop that were subsequently analyzed.

**Figure 4:**
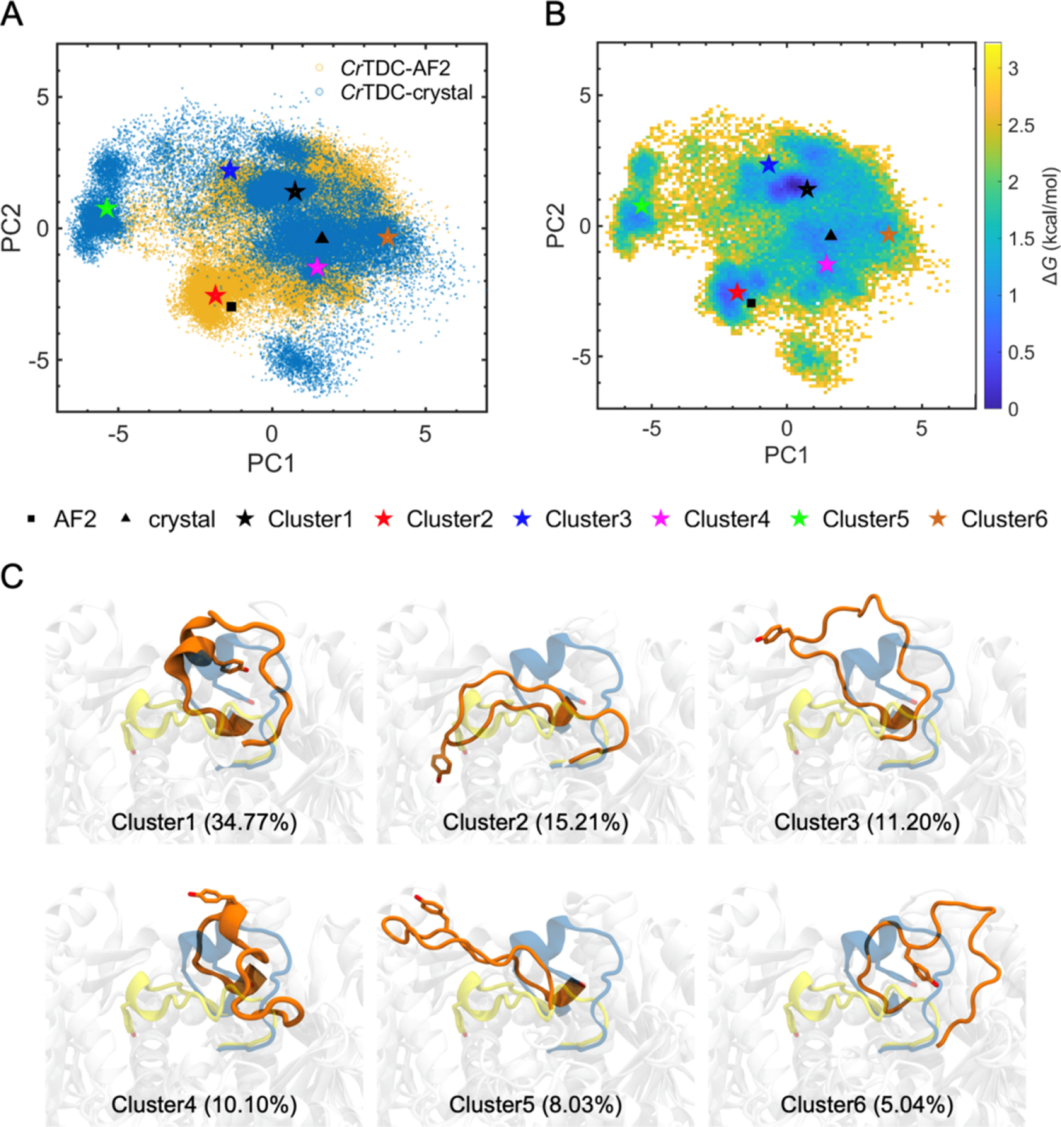
MD simulations of the *Cr*TDC-AF2 and *Cr*TDC-crystal systems. (A) Projection of the large loop movement onto the first two principal components PC1 and PC2. The yellow and blue scattered dots represent trajectories from *Cr*TDC-AF2 and *Cr*TDC-crystal simulations, while the black square and triangle indicate the initial closed (*Cr*TDC-crystal) and open (*Cr*TDC-AF2) states of the loop, respectively (pentagrams: centroid structures of the top six clusters from clustering analysis of the large loop). (B) Free energy surface over PC1 and PC2 determined from the combined *Cr*TDC-AF2 and *Cr*TDC-crystal trajectories. (C) Centroid structures of the large catalytic loop. The initial *Cr*TDC-AF2 (yellow) and *Cr*TDC-crystal (blue) structures are shown as transparent cartoon representations for reference.

**Figure 5:**
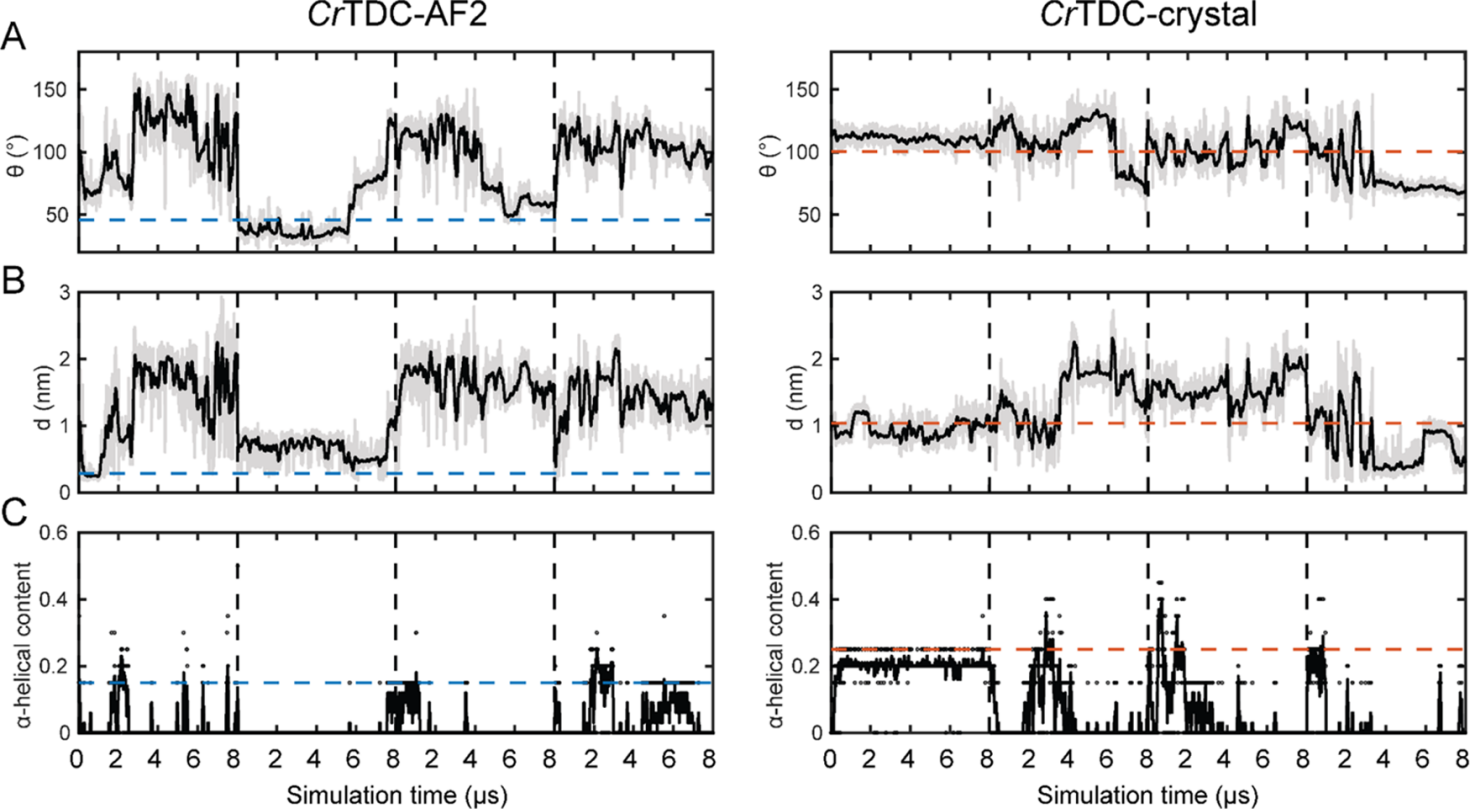
Conformation of the *Cr*TDC large loop during MD simulations initiated from *Cr*TDC-AF2 and *Cr*TDC-crystal structures. (A) θ, (B) *d*, and (C) the α-helical content of the catalytic large loop during the simulations. The horizontal dashed lines represent the corresponding values of the initial *Cr*TDC-AF2 (blue) and *Cr*TDC-crystal (orange) structures, respectively. Results from individual 8 μs simulations are merged and displayed in all panels separated by vertical dashed lines.

To quantitatively characterize conformations of the large loop in *Cr*TDC, we performed principal component analysis (PCA) on the combined simulation trajectories initiated from its crystal structure and AF2 prediction. The first two principal components (PC1 and PC2, Figure 4A,B) captured approximately 72% of the variance in loop movement, which then served as two basis vectors onto which the simulation trajectories were projected. The top six clusters from subsequent clustering analysis collectively accounted for 84% of the loop conformations, the projections of which onto PC1 and PC2 were also indicated in Figure 4A,B. To further assist the analysis of loop conformations, we computed two additional metrics, namely, the minimum distance *d* between atoms from the key residue Tyr348 and its proton transfer partner His203, as well as an opening angle θ that measured the extent to which the loop opened from the mouth of the active pocket (Figure 5A,B). Specifically, θ was defined as the angle between the vector from Leu342 to Tyr348 and the vector from Leu342 to the *z*-axis projection of residues 342–361 C_α_ atoms’ center according to the *Cr*TDC-AF2 structure (Figure S4). The Tyr348-His203 distance *d* and the loop opening angle θ therefore complement the PCA and clustering analysis in characterizing the diverse conformations sampled by the large loop during the aggregated tens of microsecond MD simulations.

Initiated from either the AF2 (black square) or the crystal structure (black triangle), the *Cr*TDC large loop explored a wide range of conformations, displaying a broad distribution over the 2D coordinate space spanned by PC1 and PC2 (Figure 4A,B). Overall, the range of conformations sampled by simulations initiated from the *Cr*TDC-AF2 structure appeared to be slightly broader than those initiated from the *Cr*TDC-crystal structure, as evidenced by the former’s wider coverage of the PC1-PC2 (Figure 4A) as well as the θ–*d* coordinate space and the individual distributions in θ and *d* (Figure S5). Their difference in these profiles is a clear indication that simulations initiated from the two states each sampled the loop conformational space to a different extent. While individually they were insufficient to sample the entire conformational space of the catalytic loop well, collectively these simulations could be used to explore characteristic structural and dynamic features of this space. As shown in Figure 4B,C, highly populated loop conformations emerged as dark regions with low free energy in the 2D PC1-PC2 space and can be identified by RMSD-based clustering analysis. Centroid structures obtained from the clustering analysis overlapped reasonably well with the low free energy regions on the PC1-PC2 map. Clusters 1 and 2, which represented ∼35% and ∼15% of the simulation trajectories, fell in the vicinity of the closed (*Cr*TDC-crystal) and open (*Cr*TDC-AF2) state of the large loop, respectively, although a clear distinction with the corresponding initial state could be found in both centroid structures. Specifically, compared to its closed state in the *Cr*TDC-AF2 structure, the large loop in cluster 2 lost the precise positioning of Tyr348 (Figure 4C), likely due to the absence of substrate and LLP. Interestingly, this cluster centroid had an even smaller θ than the initial *Cr*TDC-AF2 structure (θ = 37.7° vs 45.9°), suggesting that the loop capped the active site even more “deeply” in this conformation. Compared with the *Cr*TDC-crystal structure, cluster 1 appeared to be slightly “more open” (θ = 113.6° vs 100.3°), a difference that may have arisen from the considerable flexibility of the large loop in the apoenzyme. Such loop flexibility is consistent with our previous simulations^14^ of *Cr*TDC and has been examined in depth by a recent study on the human aromatic amino acid decarboxylase.^15^ Previous simulations on the enzyme triosephosphate isomerase have revealed that a highly flexible loop may adopt many different open states.^46^ Here, based on their locations on the 2D PC1-PC2 map as well as comparison with the *Cr*TDC-AF2 and *Cr*TDC-crystal structures (Figures 4C and S5B,C), clusters 4 and 6 appeared to represent alternative open states of the catalytic loop. In particular, the catalytic loop flipped further away from the active pocket located at the center of the homodimeric enzyme in cluster 6 (θ = 125.9°), indicative of a “wide open” conformation. As shown in Figure S5C, angle θ even reached ∼150° during simulations of both *Cr*TDC-AF2 and *Cr*TDC-crystal. Along with the broad range of conformations explored by the loop over the PC1-PC2 space, this result again reflects the remarkable flexibility of the *Cr*TDC catalytic loop and its access to multiple open (including wide open) conformations.

To evaluate the challenge posed by the vast conformational space accessible to the *Cr*TDC catalytic loop, we determined the correlation time τ of the angle θ and distance *d* from each independent replica of 8 μs MD simulations. As shown in Table S2, the large fluctuation in τ and its hundred−nanosecond mean clearly point at the challenge of sampling the loop conformation via plain MD simulations. This issue can be exacerbated by the correlation between loop secondary structure and its dynamics. As shown in Figure 4C, a small portion of the large loop (residues 346 to 350) formed a short α-helix in both cluster 1 and the *Cr*TDC-crystal structure. With the exception of cluster 4, the loop completely lost its helical content in the remaining conformations captured by clusters 2 to 6. Further analysis revealed no statistically significant difference in the average loop α-helical content between the four simulations initiated from *Cr*TDC-AF2 and those initiated from the *Cr*TDC-crystal structure (Figure 5). Nonetheless, for an individual simulation where the loop had a high α-helical content, sampling over the PC1-PC2 space was found to be markedly reduced (Figure S6), in line with the previously reported hindrance on loop movement posed by this short helix.^14^

## Discussion

Given its electron-sink nature, PLP is known to slowly catalyze many reactions in the absence of an enzyme.^1,6^ Apart from enhancing this innate catalytic power of the cofactor, a key task of all PLP-dependent enzymes is to enforce substrate and reaction-type specificities through carefully selected and positioned active-site residues. On the control of reaction type, the Dunathan hypothesis^47^ explains how the substrate orientation relative to the PLP pyridine ring determines which bond to C_α_ is broken upon formation of the external aldimine. The reaction specificity control does not end here: as the quinonoid intermediate can react in a number of possible pathways, the enzyme must continue to govern subsequent steps to promote the desired reaction while minimizing the unwanted, side ones.^6,26^ Different strategies may be devised, depending on the competing reactions at play. For instance, alanine racemase has been shown to selectively destabilize the quinonoid intermediate to promote racemization over the transamination side reaction.^8^ For the mammalian DDC, O_2_-involved oxidation is known to be a side reaction.^48,49^ In plants, however, AASs have evolved to fully couple decarboxylation with oxidative deamination, making it their primary reaction instead.^5,9−11^ A number of factors, including the ease of O_2_ access,^5,14^ have been hypothesized to play a role in the above switch of reaction-type specificity between the two categories of closely related enzymes. The experimentally demonstrated conversion of a canonical plant AAAD to an AAS upon a tyrosine to phenylalanine mutation (and vice versa) further focuses one’s attention on the impact of this single amino acid switch.

Our ILS calculations of the closed-state *Ps*TyDC reveal O_2_ migration pathways in both the WT and Y350F mutant protein, allowing for detailed analysis of oxygen accessibility to their active sites. The difference in the free energy cost of the entrance of the O_2_ into WT *Ps*TyDC and its Y350F mutant is found to be statistically insignificant. Furthermore, the barrier heights in both proteins are comparable with the thermal energy, indicating that their active sites can be readily accessed by a diffusing oxygen molecule. These results suggest that oxygen accessibility is unlikely a significant contributor to different reaction-type specificities of the canonical plant AAADs and AASs. In other words, the dominance of decarboxylation over oxidation in WT *Ps*TyDC or vice versa in the Y350F mutant does not simply arise from their distinction in O_2_ accessibility. The conversion of a canonical AAAD to AAS upon the Tyr-to-Phe mutation can therefore be primarily attributed to the proton-donating ability of the former amino acid and the lack of such an ability of the latter: replacing the tyrosine by phenylalanine leaves the quinonoid intermediate without a proton donor, giving the oxygen molecule an opportunity to attack and direct the reaction toward oxidative deamination. Based on the ease of the gas molecule entering the *Ps*TyDC active site revealed by our ILS calculations, a competition between protonation by Tyr350 and oxidation by O_2_ should always be present once the quinonoid intermediate is produced. This suggests that the former reaction is likely faster than the latter and therefore kinetically favored by the WT *Ps*TyDC. Such a difference would be consistent with kinetic measurement in other plant canonical AAADs and AASs: on the one hand, the Y348F *Cr*TDC mutant is 17 times slower in its aldehyde synthase activity than the decarboxylase activity of the WT protein; on the other hand, the F338Y mutant of *At*PAAS is found to be 3-fold faster as a converted decarboxylase than the original, WT aldehyde synthase.^11^ Mechanistically, oxygen consumption by PLP-dependent enzymes has yet to be fully understood. The normal, triplet state of O_2_ is more stable than its singlet state, and enzymes that take oxygen as a substrate typically employ transition state metal-containing cofactors or organic redox cofactors such as flavin. While the reactivity of the quinonoid intermediate with oxygen has been demonstrated in DDC,^50,51^ exactly how AASs promote decarboxylation-dependent oxidative deamination as their primary reaction and chemical details following the decarboxylation step await further investigation.

Structural information on plant AAADs has opened the door to employ MD simulations to explore their conformational dynamics. With microsecond simulations initiated from both the open-state crystal structure and the closed-state AF2 model, we set out to uncover the dynamics of the *Cr*TDC large catalytic loop not captured by previous hundreds of nanoseconds simulations initiated from the crystal structure alone. Apart from the open and closed states, as revealed by the crystal structure and AF2 prediction, these simulations uncover a wide range of loop conformations. Loop movement, as measured by a characteristic angle θ and the distance *d* between the key partners Tyr348 and His203, has an average correlation time of up to ∼500 ns. While similar loop dynamics may be expected for *Ps*TyDC given the indispensable role played by the conserved Tyr, sequence variation of the catalytic loop between these two enzymes may produce conformational and/or secondary structural differences, which remains to be investigated by future studies. Mechanistically, given that decarboxylases have a low to moderate turnover rate on the order of 1–10 s^–1^,^5,12^ representative of an “average” enzyme,^52^ loop movement with the above correlation time may still be far removed from being rate limiting. However, such a correlation time already presents a significant challenge to MD modeling, suggesting that tens of microseconds or even longer simulations may be needed to fully sample an AAAD’s loop conformational space. This challenge is neither new nor uncommon, as loop sampling in other enzymes has been shown to be computationally demanding.^46,53,54^ As a result, apart from multiple replicas of simulations sufficiently longer than the correlation time of loop movement, enhanced sampling approaches^53,55,56^ should be explored in future studies of the plant AAAD family.

## Conclusions

In this work, we examined key structural and dynamic elements underlying the differential enzymatic functions of plant AAADs through a combination of modeling approaches. Our implicit ligand sampling calculations on *Ps*TyDC revealed similar oxygen accessibility in both the wild-type enzyme and its Y350F mutant. The calculated 3D O_2_ free energy profiles identify two major migration pathways and negligible barriers for oxygen entrance into both the WT and mutant enzymes, suggesting that a competition between the straightforward decarboxylation and decarboxylation-dependent oxidative deamination is always present in their active sites, and the dominance of one reaction type over the other cannot be attributed to oxygen accessibility. Along with previous experiments,^11^ these results strongly indicate that the ability to donate a proton to the quinonoid reaction intermediate by the conserved tyrosine in canonical AAADs and the lack of such an ability by the corresponding phenylalanine in AASs predominantly underlies the divergence in catalytic pathways of the two types of enzymes from the same AAAD family. Through microsecond MD simulations on *Cr*TDC initiated from its open-state crystal structure and closed-state AF2 prediction, we extensively examined the intricate conformational and dynamic details of its large loop housing the key catalytic tyrosine residue. The correlation time of loop movement highlights the challenge to conventional MD and the need for enhanced sampling approaches to comprehensively explore the conformational space of this indispensable catalytic loop of plant AAADs.

## Supporting Information Available

O_2_ free energy (Table S1); correlation time τ for θ and *d* (Table S2); chemical structures of LLP and external aldimine (Figure S1); MD simulation systems (Figure S2); 3D water occupancy (Figure S3); definition of θ (Figure S4); scatter plot (Figure S5), and secondary structure of CrTDC large loop (Figure S6) (PDF)

## References

[ref1] Liang J.; Han Q.; Tan Y.; Ding H.; Li J. Current Advances on Structure-Function Relationships of Pyridoxal 5′-Phosphate-Dependent Enzymes. Front Mol. Biosci. 2019, 6, 4 10.3389/fmolb.2019.00004. 30891451 PMC6411801

[ref2] Han S.-W.; Shin J.-S. Aromatic L-amino acid decarboxylases: mechanistic features and microbial applications. Appl. Microbiol. Biotechnol. 2022, 106, 4445–4458. 10.1007/s00253-022-12028-4. 35763068

[ref3] Facchini P. J.; Huber-Allanach K. L.; Tari L. W. Plant aromatic L-amino acid decarboxylases: evolution, biochemistry, regulation, and metabolic engineering applications. Phytochemistry 2000, 54, 121–138. 10.1016/S0031-9422(00)00050-9. 10872203

[ref4] Teoh E. S. Secondary Metabolites of Plants. In Medicinal Orchids of Asia; Springer International Publishing: Cham, 2016; pp 59–73.

[ref5] Kaminaga Y.; Schnepp J.; Peel G.; Kish C. M.; Ben-Nissan G.; Weiss D.; Orlova I.; Lavie O.; Rhodes D.; Wood K.; et al. Plant phenylacetaldehyde synthase is a bifunctional homotetrameric enzyme that catalyzes phenylalanine decarboxylation and oxidation. J. Biol. Chem. 2006, 281, 23357–23366. 10.1074/jbc.M602708200. 16766535

[ref6] Eliot A. C.; Kirsch J. F. Pyridoxal phosphate enzymes: mechanistic, structural, and evolutionary considerations. Annu. Rev. Biochem. 2004, 73, 383–415. 10.1146/annurev.biochem.73.011303.074021. 15189147

[ref7] Hoegl A.; Nodwell M. B.; Kirsch V. C.; Bach N. C.; Pfanzelt M.; Stahl M.; Schneider S.; Sieber S. A. Mining the cellular inventory of pyridoxal phosphate-dependent enzymes with functionalized cofactor mimics. Nat. Chem. 2018, 10, 1234–1245. 10.1038/s41557-018-0144-2. 30297752 PMC6252082

[ref8] Toney M. D. Reaction specificity in pyridoxal phosphate enzymes. Arch. Biochem. Biophys. 2005, 433, 279–287. 10.1016/j.abb.2004.09.037. 15581583

[ref9] Torrens-Spence M. P.; Gillaspy G.; Zhao B.; Harich K.; White R. H.; Li J. Biochemical evaluation of a parsley tyrosine decarboxylase results in a novel 4-hydroxyphenylacetaldehyde synthase enzyme. Biochem. Biophys. Res. Commun. 2012, 418, 211–216. 10.1016/j.bbrc.2011.12.124. 22266321

[ref10] Torrens-Spence M. P.; von Guggenberg R.; Lazear M.; Ding H.; Li J. Diverse functional evolution of serine decarboxylases: identification of two novel acetaldehyde synthases that uses hydrophobic amino acids as substrates. BMC Plant Biol. 2014, 14, 247 10.1186/s12870-014-0247-x. 25230835 PMC4177580

[ref11] Torrens-Spence M. P.; Liu P.; Ding H.; Harich K.; Gillaspy G.; Li J. Biochemical evaluation of the decarboxylation and decarboxylation-deamination activities of plant aromatic amino acid decarboxylases. J. Biol. Chem. 2013, 288, 2376–2387. 10.1074/jbc.M112.401752. 23204519 PMC3554908

[ref12] Bertoldi M.; Gonsalvi M.; Contestabile R.; Voltattorni C. B. Mutation of tyrosine 332 to phenylalanine converts dopa decarboxylase into a decarboxylation-dependent oxidative deaminase. J. Biol. Chem. 2002, 277, 36357–36362. 10.1074/jbc.M204867200. 12118007

[ref13] Takeshima D.; Mori A.; Ito H.; Komori H.; Ueno H.; Nitta Y. A single amino acid substitution converts a histidine decarboxylase to an imidazole acetaldehyde synthase. Arch. Biochem. Biophys. 2020, 693, 108551 10.1016/j.abb.2020.108551. 32871134

[ref14] Torrens-Spence M. P.; Chiang Y. C.; Smith T.; Vicent M. A.; Wang Y.; Weng J. K. Structural basis for divergent and convergent evolution of catalytic machineries in plant aromatic amino acid decarboxylase proteins. Proc. Natl. Acad. Sci. U.S.A. 2020, 117, 10806–10817. 10.1073/pnas.1920097117. 32371491 PMC7245119

[ref15] Bisello G.; Ribeiro R. P.; Perduca M.; Belviso B. D.; De' Laureto P. P.; Giorgetti A.; Caliandro R.; Bertoldi M. Human aromatic amino acid decarboxylase is an asymmetric and flexible enzyme: Implication in aromatic amino acid decarboxylase deficiency. Protein Sci. 2023, 32, e4732 10.1002/pro.4732. 37466248 PMC10382914

[ref16] Jumper J.; Evans R.; Pritzel A.; Green T.; Figurnov M.; Ronneberger O.; Tunyasuvunakool K.; Bates R.; Zidek A.; Potapenko A.; Bridgland A.; Meyer C.; Kohl S. A. A.; Ballard A. J.; Cowie A.; Romera-Paredes B.; Nikolov S.; Jain R.; Adler J.; Back T.; Petersen S.; Reiman D.; Clancy E.; Zielinski M.; Steinegger M.; Pacholska M.; Berghammer T.; Bodenstein S.; Silver D.; Vinyals O.; Senior A. W.; Kavukcuoglu K.; Kohli P.; Hassabis D. Highly accurate protein structure prediction with AlphaFold. Nature 2021, 596, 583–589. 10.1038/s41586-021-03819-2. 34265844 PMC8371605

[ref17] Varadi M.; Anyango S.; Deshpande M.; Nair S.; Natassia C.; Yordanova G.; Yuan D.; Stroe O.; Wood G.; Laydon A.; Zidek A.; Green T.; Tunyasuvunakool K.; Petersen S.; Jumper J.; Clancy E.; Green R.; Vora A.; Lutfi M.; Figurnov M.; Cowie A.; Hobbs N.; Kohli P.; Kleywegt G.; Birney E.; Hassabis D.; Velankar S. AlphaFold Protein Structure Database: massively expanding the structural coverage of protein-sequence space with high-accuracy models. Nucleic Acids Res. 2022, 50, D439–D444. 10.1093/nar/gkab1061. 34791371 PMC8728224

[ref18] Søndergaard C. R.; Olsson M. H. M.; Rostkowski M.; Jensen J. H. Improved Treatment of Ligands and Coupling Effects in Empirical Calculation and Rationalization of pKa Values. J. Chem. Theory Comput. 2011, 7, 2284–2295. 10.1021/ct200133y. 26606496

[ref19] Olsson M. H. M.; Søndergaard C. R.; Rostkowski M.; Jensen J. H. PROPKA3: Consistent Treatment of Internal and Surface Residues in Empirical pKa Predictions. J. Chem. Theory Comput. 2011, 7, 525–537. 10.1021/ct100578z. 26596171

[ref20] DeLano W. L. Pymol: An open-source molecular graphics tool. CCP4 Newsl. Protein Crystallogr. 2002, 40, 82–92.

[ref21] Phillips J. C.; Hardy D. J.; Maia J. D. C.; Stone J. E.; Ribeiro J. V.; Bernardi R. C.; Buch R.; Fiorin G.; Hénin J.; Jiang W.; et al. Scalable molecular dynamics on CPU and GPU architectures with NAMD. J. Chem. Phys. 2020, 153, 044130 10.1063/5.0014475. 32752662 PMC7395834

[ref22] Huang J.; Rauscher S.; Nawrocki G.; Ran T.; Feig M.; de Groot B. L.; Grubmuller H.; MacKerell A. D. Jr. CHARMM36m: an improved force field for folded and intrinsically disordered proteins. Nat. Methods 2017, 14, 71–73. 10.1038/nmeth.4067. 27819658 PMC5199616

[ref23] Chan-Huot M.; Dos A.; Zander R.; Sharif S.; Tolstoy P. M.; Compton S.; Fogle E.; Toney M. D.; Shenderovich I.; Denisov G. S.; Limbach H.-H. NMR studies of protonation and hydrogen bond states of internal aldimines of pyridoxal 5′-phosphate acid-base in alanine racemase, aspartate aminotransferase, and poly-L-lysine. J. Am. Chem. Soc. 2013, 135, 18160–18175. 10.1021/ja408988z. 24147985

[ref24] Caulkins B. G.; Bastin B.; Yang C.; Neubauer T. J.; Young R. P.; Hilario E.; Huang Y.-M. M.; Chang C.-E. A.; Fan L.; Dunn M. F.; Marsella M. J.; Mueller L. J. Protonation states of the tryptophan synthase internal aldimine active site from solid-state NMR spectroscopy: direct observation of the protonated Schiff base linkage to pyridoxal-5′-phosphate. J. Am. Chem. Soc. 2014, 136, 12824–12827. 10.1021/ja506267d. 25148001 PMC4183654

[ref25] Sharif S.; Denisov G. S.; Toney M. D.; Limbach H.-H. NMR studies of coupled low- and high-barrier hydrogen bonds in pyridoxal-5′-phosphate model systems in polar solution. J. Am. Chem. Soc. 2007, 129, 6313–6327. 10.1021/ja070296+. 17455937

[ref26] Toney M. D. Aspartate aminotransferase: an old dog teaches new tricks. Arch. Biochem. Biophys. 2014, 544, 119–127. 10.1016/j.abb.2013.10.002. 24121043 PMC3946379

[ref27] Vanommeslaeghe K.; MacKerell A. D. Jr. Automation of the CHARMM General Force Field (CGenFF) I: bond perception and atom typing. J. Chem. Inf. Model. 2012, 52, 3144–3154. 10.1021/ci300363c. 23146088 PMC3528824

[ref28] Vanommeslaeghe K.; Hatcher E.; Acharya C.; Kundu S.; Zhong S.; Shim J.; Darian E.; Guvench O.; Lopes P.; Vorobyov I.; Mackerell A. D. Jr. CHARMM general force field: A force field for drug-like molecules compatible with the CHARMM all-atom additive biological force fields. J. Comput. Chem. 2010, 31, 671–690. 10.1002/jcc.21367. 19575467 PMC2888302

[ref29] Vanommeslaeghe K.; Raman E. P.; MacKerell A. D. Jr. Automation of the CHARMM General Force Field (CGenFF) II: assignment of bonded parameters and partial atomic charges. J. Chem. Inf. Model. 2012, 52, 3155–3168. 10.1021/ci3003649. 23145473 PMC3528813

[ref30] Mayne C. G.; Saam J.; Schulten K.; Tajkhorshid E.; Gumbart J. C. Rapid parameterization of small molecules using the Force Field Toolkit. J. Comput. Chem. 2013, 34, 2757–2770. 10.1002/jcc.23422. 24000174 PMC3874408

[ref31] Darden T.; York D.; Pedersen L. Particle mesh Ewald: An N· log (N) method for Ewald sums in large systems. J. Chem. Phys. 1993, 98, 10089–10092. 10.1063/1.464397.

[ref32] Essmann U.; Perera L.; Berkowitz M. L.; Darden T.; Lee H.; Pedersen L. G. A smooth particle mesh Ewald method. J. Chem. Phys. 1995, 103, 8577–8593. 10.1063/1.470117.

[ref33] Nosé S. A molecular dynamics method for simulations in the canonical ensemble. Mol. Phys. 1984, 52, 255–268. 10.1080/00268978400101201.

[ref34] Hoover W. G. Canonical dynamics: Equilibrium phase-space distributions. Phys. Rev. A 1985, 31, 1695 10.1103/PhysRevA.31.1695. 9895674

[ref35] Abraham M. J.; Murtola T.; Schulz R.; Páll S.; Smith J. C.; Hess B.; Lindahl E. GROMACS: High performance molecular simulations through multi-level parallelism from laptops to supercomputers. SoftwareX 2015, 1–2, 19–25. 10.1016/j.softx.2015.06.001.

[ref36] Bussi G.; Donadio D.; Parrinello M. Canonical sampling through velocity rescaling. J. Chem. Phys. 2007, 126, 014101 10.1063/1.2408420. 17212484

[ref37] Bernetti M.; Bussi G. Pressure control using stochastic cell rescaling. J. Chem. Phys. 2020, 153, 114107 10.1063/5.0020514. 32962386

[ref38] Hess B.; Bekker H.; Berendsen H. J. C.; Fraaije J. G. LINCS: A linear constraint solver for molecular simulations. J. Comput. Chem. 1997, 18, 1463–1472. 10.1002/(SICI)1096-987X(199709)18:12<1463::AID-JCC4>3.0.CO;2-H.

[ref39] Hess B. P-LINCS: A parallel linear constraint solver for molecular simulation. J. Chem. Theory Comput. 2008, 4, 116–122. 10.1021/ct700200b. 26619985

[ref40] Humphrey W.; Dalke A.; Schulten K. VMD: Visual molecular dynamics. J. Mol. Graphics 1996, 14, 33–38. 10.1016/0263-7855(96)00018-5. 8744570

[ref41] Kabsch W.; Sander C. Dictionary of protein secondary structure: pattern recognition of hydrogen-bonded and geometrical features. Biopolymers 1983, 22, 2577–2637. 10.1002/bip.360221211. 6667333

[ref42] Touw W. G.; Baakman C.; Black J.; te Beek T. A. H.; Krieger E.; Joosten R. P.; Vriend G. A series of PDB-related databanks for everyday needs. Nucleic Acids Res. 2015, 43, D364–D368. 10.1093/nar/gku1028. 25352545 PMC4383885

[ref43] Cohen J.; Arkhipov A.; Braun R.; Schulten K. Imaging the migration pathways for O2, CO, NO, and Xe inside myoglobin. Biophys. J. 2006, 91, 1844–1857. 10.1529/biophysj.106.085746. 16751246 PMC1544290

[ref44] Cohen J.; Olsen K. W.; Schulten K. Finding Gas Migration Pathways in Proteins Using Implicit Ligand Sampling. In Methods in Enzymology; Poole R. K., Ed.; Academic Press, 2008; pp 439–457. 10.1016/S0076-6879(07)37022-5 18433641

[ref45] Scharlin P.; Battino R.; Silla E.; Tucyn I.; Pascual-Ahuir J. L. Solubility of gases in water: correlation between solubility and the number of water molecules in the first solvation shell. Pure Appl. Chem. 1998, 70 (10), 1895–1904. 10.1351/pac199870101895.

[ref46] Liao Q.; Kulkarni Y.; Sengupta U.; Petrović D.; Mulholland A. J.; van der Kamp M. W.; Strodel B.; Kamerlin S. C. L. Loop Motion in Triosephosphate Isomerase Is Not a Simple Open and Shut Case. J. Am. Chem. Soc. 2018, 140, 15889–15903. 10.1021/jacs.8b09378. 30362343

[ref47] Dunathan H. C. Conformation and reaction specificity in pyridoxal phosphate enzymes. Proc. Natl. Acad. Sci. U.S.A. 1966, 55, 712–716. 10.1073/pnas.55.4.712. 5219675 PMC224217

[ref48] Bertoldi M.; Moore P. S.; Maras B.; Dominici P.; Voltattorni C. B. Mechanism-based inactivation of dopa decarboxylase by serotonin. J. Biol. Chem. 1996, 271, 23954–23959. 10.1074/jbc.271.39.23954. 8798628

[ref49] Bertoldi M.; Dominici P.; Moore P. S.; Maras B.; Voltattorni C. B. Reaction of dopa decarboxylase with alpha-methyldopa leads to an oxidative deamination producing 3,4-dihydroxyphenylacetone, an active site directed affinity label. Biochemistry 1998, 37, 6552–6561. 10.1021/bi9718898. 9572873

[ref50] Bertoldi M.; Cellini B.; Montioli R.; Voltattorni C. B. Insights into the Mechanism of Oxidative Deamination Catalyzed by DOPA Decarboxylase. Biochemistry 2008, 47, 7187–7195. 10.1021/bi800478s. 18547057

[ref51] Bisello G.; Longo C.; Rossignoli G.; Phillips R. S.; Bertoldi M. Oxygen reactivity with pyridoxal 5′-phosphate enzymes: biochemical implications and functional relevance. Amino Acids 2020, 52, 1089–1105. 10.1007/s00726-020-02885-6. 32844248 PMC7497351

[ref52] Bar-Even A.; Noor E.; Savir Y.; Liebermeister W.; Davidi D.; Tawfik D. S.; Milo R. The moderately efficient enzyme: evolutionary and physicochemical trends shaping enzyme parameters. Biochemistry 2011, 50, 4402–4410. 10.1021/bi2002289. 21506553

[ref53] Romero-Rivera A.; Corbella M.; Parracino A.; Patrick W. M.; Kamerlin S. C. L. Complex Loop Dynamics Underpin Activity, Specificity, and Evolvability in the (βα)8 Barrel Enzymes of Histidine and Tryptophan Biosynthesis. JACS Au 2022, 2, 943–960. 10.1021/jacsau.2c00063. 35557756 PMC9088769

[ref54] Crean R. M.; Biler M.; van der Kamp M. W.; Hengge A. C.; Kamerlin S. C. L. Loop Dynamics and Enzyme Catalysis in Protein Tyrosine Phosphatases. J. Am. Chem. Soc. 2021, 143, 3830–3845. 10.1021/jacs.0c11806. 33661624 PMC8031367

[ref55] Konovalov K. A.; Unarta I. C.; Cao S.; Goonetilleke E. C.; Huang X. Markov State Models to Study the Functional Dynamics of Proteins in the Wake of Machine Learning. JACS Au 2021, 1, 1330–1341. 10.1021/jacsau.1c00254. 34604842 PMC8479766

[ref56] Hénin J.; Lelièvre T.; Shirts M. R.; Valsson O.; Delemotte L. Enhanced sampling methods for molecular dynamics simulations. Living J. Comput. Mol. Sci. 2022, 4, 1583 10.33011/livecoms.4.1.1583.

