# Supplementary material for: Active-Site Oxygen Accessibility and Catalytic Loop Dynamics of Plant Aromatic Amino Acid Decarboxylases from Molecular Simulations
Source: Biochemistry. 2024 Jul 15;63(15):1980–90. doi: 10.1021/acs.biochem.4c00144 (PMC11308512; doi:10.1021/acs.biochem.4c00144)
Supplement: Supplementary file 1 — bi4c00144_si_001.pdf [file bi4c00144_si_001.pdf]

Supporting Information

Active-site Oxygen Accessibility and Catalytic Loop Dynamics of Plant Aromatic Amino Acid Decarboxylases from Molecular Simulations

Yitao Gou, Tianjie Li, Yi Wang*

Department of Physics, The Chinese University of Hong Kong, Shatin, New Territories, Hong Kong, China

*Correspondence: yiwang@cuhk.edu.hk

number of pages	8
number of figures	6
number of tables	2

Table S1. O_2 free energy (mean ± standard deviation) obtained from ILS calculations based on simulations of apo- or two holo-forms of *Ps*TyDC in complex with either LLP and substrate, or, the external aldimine. Results obtained from all simulations of a given protein (WT or the Y350F mutant) or bulk water are listed in the last column.

	Apo (n=10)	Holo (LLP+substrate) (n=10)	Holo (external aldimine) (n=10)	All (n=30)
WT	3.20±1.13	2.97±1.18	2.83±0.95	2.94±1.02
Y350F	2.54±0.87	2.63±0.85	2.72±1.38	2.70±1.06
Water	2.06±0.02	2.05±0.03	2.04±0.02	2.05±0.02

Table S2. The correlation time τ for θ and *d*.

system	*Cr*TDC-AF2				*Cr*TDC-crystal				Mean±std
	1	2	3	4	1	2	3	4	
τ (θ) (ns)	528.4	813.6	1053.1	77.6	223.0	467.8	307.3	649.1	515.0±320.5
τ (*d*) (ns)	603.2	210.8	69.9	148.8	191.4	695.6	229.3	356.0	313.1±223.8

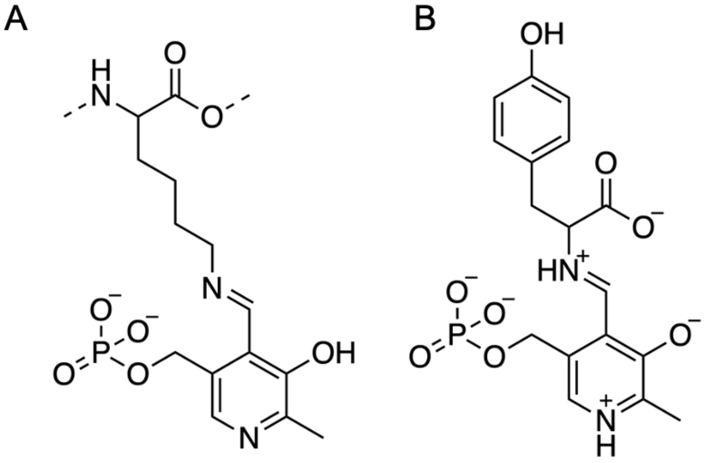

Figure S1. Chemical structures of LLP (A) and external aldimine (B) used in the holo-simulations of *Ps*TyDC. Dashed lines indicate the connection of LLP to neighboring amino acids.

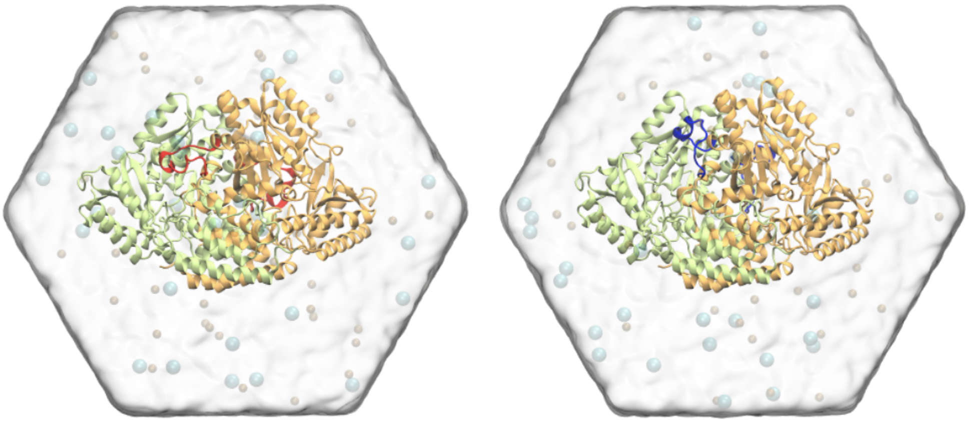

Figure S2. MD simulation systems of *Cr*TDC-AF2 (left) and *Cr*TDC-crystal (right). The two structures are identical except for the large catalytic loop (Res 342-361). The dimeric protein is colored in yellow (monomer A) and green (monomer B), with the catalytic loop colored in red for *Cr*TDC-AF2 and blue for *Cr*TDC-crystal, respectively. The neutralizing ions are shown in transparent spheres with Na+ in ochre and Cl- in cyan. The dodecahedron water boxes are shown in white transparent surfaces.

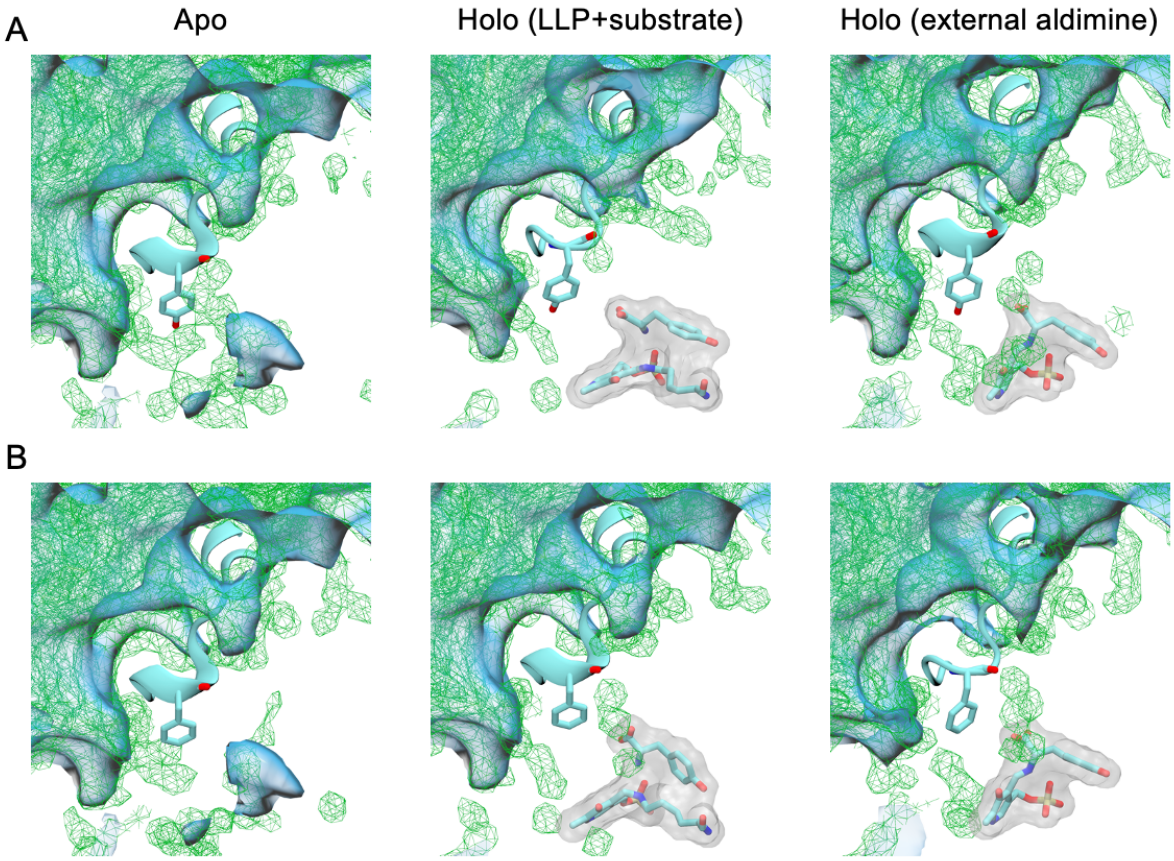

Figure S3. 3D water occupancy of the WT *Ps*TyDC (A) and its Y350F mutant (B). The green wireframes represent isosurfaces with an isovalue of 0.31, which is the same as average water occupancy measured for bulk solution at a grid spacing of 1 Å. The residue Tyr350 or its Phe350 mutant, LLP and the substrate as well as the external aldimine are highlighted.

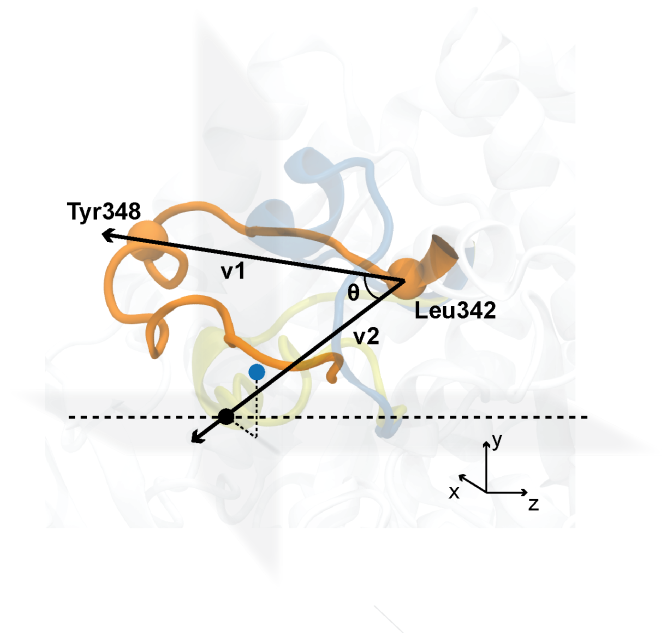

Figure S4. Schematic diagram showing the definition of θ as the angle between two vectors v_1 and v_2: v_1 is defined as the vector pointing from Leu342 $C_α$ atom to Tyr348 $C_α$ atom; v_2 is defined as the vector pointing from Leu342 $C_α$ atom to a constant position in space, namely, the center of residues 342-361 $C_α$ atoms in the *Cr*TDC-AF2 structure (blue dot) projected onto the z-axis (black dot). Prior to the calculation of θ, the long axis (the third principal axis obtained with the VMD *orient* plugin) of the *Cr*TDC homodimer is aligned by the *z* axis (dashed line) and its center of mass is placed at the origin.

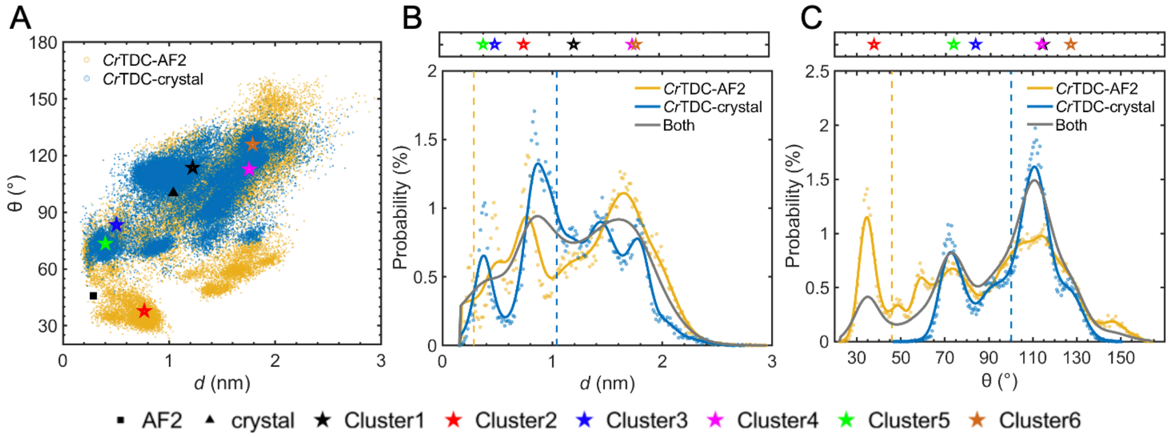

Figure S5. (A) Scatter plot over the 2D θ-*d* space. The yellow and blue circles correspond to trajectories of *Cr*TDC-AF2 and *Cr*TDC-crystal simulations, respectively. The black square and triangle denote the initial *Cr*TDC-AF2 and *Cr*TDC-crystal structures. The pentagrams represent the centroid structures of top six clusters from RMSD-based clustering analysis of the large loop C_α atoms. (B,C) The probability distribution of *d* (B) and θ (C). Scattered circles show the raw data from simulations initiated from *Cr*TDC-AF2 and *Cr*TDC-crystal, while the solid curves represent smoothed data using Gaussian-weighted average. Different colors show the corresponding values from different initial structures, specifically, orange (*Cr*TDC-AF2), blue (*Cr*TDC-crystal) and gray (both *Cr*TDC-AF2 and *Cr*TDC-crystal). Orange and blue dashed lines indicate corresponding values obtained from the *Cr*TDC-AF2 and *Cr*TDC-crystal structures.

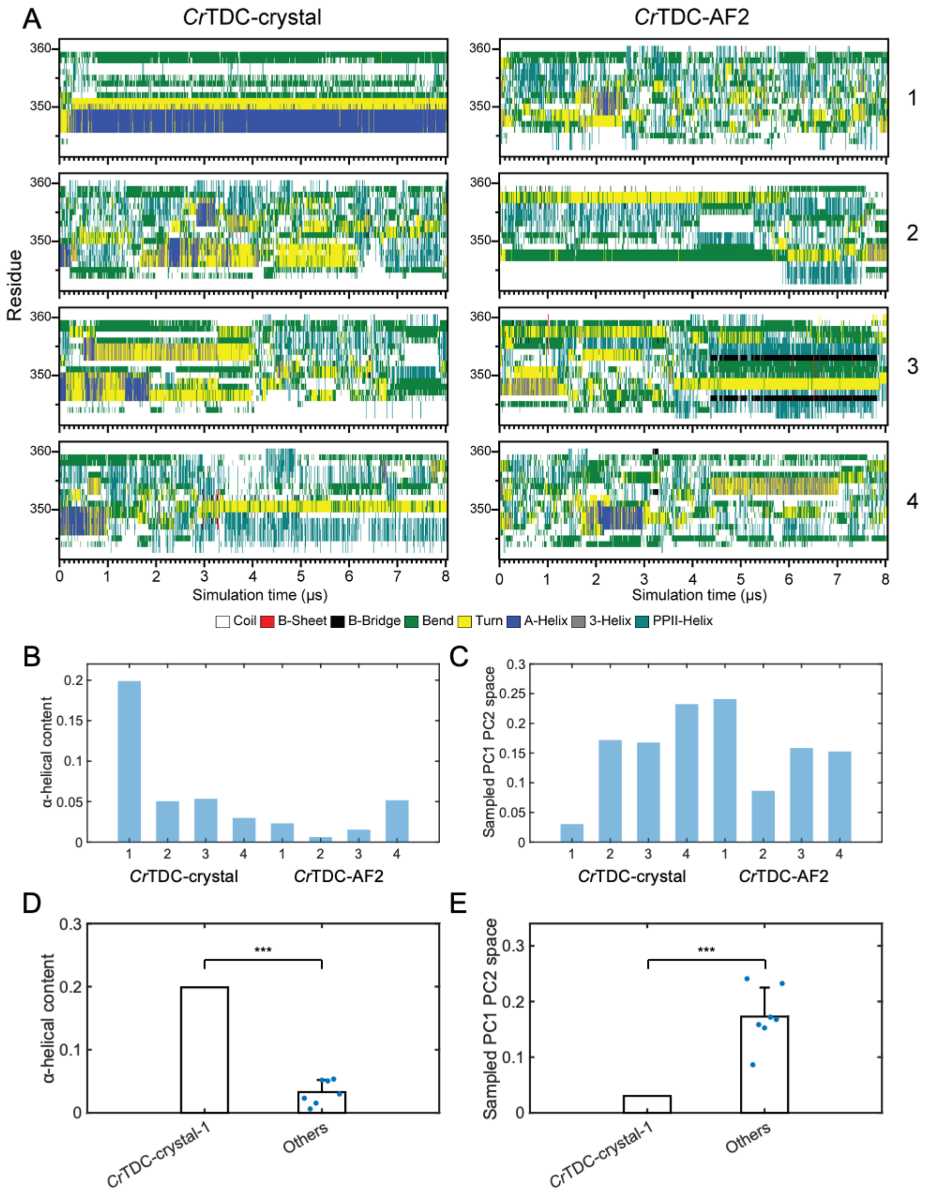

Figure S6. (A) Secondary structure of *Cr*TDC large loop computed using the DSSP program (see Methods). (B,C) The average α-helical content (B) and sampled PC1-PC2 space (C) of the catalytic large loop in each simulated monomer. With the two monomers in a *Cr*TDC dimer treated separately, two replicas of simulations initiated from either *Cr*TDC-crystal or *Cr*TDC-AF2 each resulted in four simulated monomers. The percentage of grid points occupied by a given simulated monomer on the 2D PC1-PC2 map is taken as its sampled PC1-PC2 space. (D,E) The comparison of the α-helical content (D) and the sampled PC1-PC2 space (E) between *Cr*TDC-crystal-1 and other systems. The difference between the simulated monomer CrTDC-crystal-1 and others has a statistical significance at the 0.001 level (denoted by ***) in both α-helical content and sampled PC1-PC2 space.
